# Accelerated Non-Contrast-Enhanced Three-Dimensional Cardiovascular Magnetic Resonance Deep Learning Reconstruction

**DOI:** 10.31083/RCM37399

**Published:** 2025-07-22

**Authors:** Sukran Erdem, Orhan Erdem, M. Tarique Hussain, F. Gerald Greil, Qing Zou

**Affiliations:** ^1^Division of Pediatric Cardiology, Department of Pediatrics, The University of Texas Southwestern Medical Center, Dallas, TX 75235, USA; ^2^Department of Advanced Data Analytics, University of North Texas, Denton, TX 76205, USA; ^3^Department of Radiology, The University of Texas Southwestern Medical Center, Dallas, TX 75235, USA; ^4^Advanced Imaging Research Center, The University of Texas Southwestern Medical Center, Dallas, TX 75235, USA

**Keywords:** Adaptive CS-Net, artificial intelligence, deep learning, cardiovascular magnetic resonance, congenital heart disease

## Abstract

**Background:**

Cardiovascular magnetic resonance (CMR) is a time-consuming, yet critical imaging method. In contrast, while rapid techniques accelerate image acquisition, these methods can also compromise image quality. Meanwhile, the effectiveness of Adaptive CS-Net, a vendor-supported deep-learning magnetic resonance (MR) reconstruction algorithm, for non-contrast three-dimensional (3D) whole-heart imaging using relaxation-enhanced angiography without contrast and triggering (REACT) remains uncertain.

**Methods:**

Thirty participants were prospectively recruited for this study. Each underwent non-contrast imaging that included a modified REACT sequence and a standard 3D balanced steady-state free precession (bSSFP) sequence. The REACT data were acquired through six-fold undersampling and reconstructed offline using both conventional compressed sensing (CS) and an Adaptive CS-Net algorithm. Subjective and objective image quality assessments, as well as cross-sectional area measurements of selected vessels, were conducted to compare the REACT images reconstructed using Adaptive CS-Net against those reconstructed using conventional CS, as well as the standard bSSFP sequence. For a statistical comparison of image quality across these three image sets, the nonparametric Friedman test was performed, followed by Dunn's post-hoc test.

**Results:**

The Adaptive CS-Net and CS-reconstructed REACT images exhibited superior image quality for pulmonary veins, neck, and upper thoracic vessels compared to the standard 3D bSSFP sequence. Adaptive CS-Net and CS reconstructed REACT images displayed significantly higher contrast-to-noise ratio (CNR) compared to those reconstructed using the 3D bSSFP sequence (all *p*-values < 0.05) for the left upper (5.40, 5.53, 0.97), left lower (6.33, 5.84, 2.27), right upper (5.49, 6.74, 1.18), and right lower pulmonary veins (6.71, 6.41, 1.26). Additionally, REACT methods showed a statistically significant improvement in CNR for both the ascending aorta and superior vena cava compared to the 3D bSSFP sequence.

**Conclusions:**

The Adaptive CS-Net reconstruction for the REACT images consistently delivered superior or comparable image quality compared to the CS technique. Notably, the Adaptive CS-Net reconstruction provides significantly enhanced image quality for pulmonary veins, neck, and upper thoracic vessels compared to 3D bSSFP.

## 1. Introduction

Cardiovascular magnetic resonance (CMR) is a gold-standard noninvasive imaging technique for evaluating heart structures and functions [1, 2]. Recent technological advances and growing concerns over the potential risks of contrast agents have led to increased interest in non-contrast-enhanced magnetic resonance imaging (MRI) techniques. Among these balanced steady-state free precession (bSSFP) sequence has been the most widely used, and available non-contrast magnetic resonance (MR) technique for CMR [3, 4]. Isotropic three-dimensional (3D) bSSFP is a reliable CMR sequence in assessing complex congenital heart disease (CHD) in infants, young children, and adults [5, 6]. However, its sensitivity to field inhomogeneities, particularly over large fields of view and high magnetic field strength, limits its use [7].

To address these limitations, newer non-contrast techniques such as relaxation-enhanced angiography without contrast and triggering (REACT) have been introduced [8, 9]. REACT employs a 3D magnetization-prepared dual-echo modified Dixon sequence to suppress static background tissue and generate high blood-to-tissue contrast over a wide field of view, without requiring image subtraction [10]. This flow-independent technique allows for multiplanar reformatting and, although originally developed for non-gated and free-breathing acquisition, can also incorporate cardiac and respiratory gating.

Nevertheless, like other MRI techniques, REACT is time-intensive, which can pose challenges in pediatric imaging. To accelerate image acquisition, methods such as parallel imaging and compressed sensing (CS) have been utilized. However, higher undersampling rates often degrade image quality. Adaptive CS-Net, a deep learning (DL)-based reconstruction algorithm developed for general MRI applications by an MR vendor (Philips Healthcare, Best, Netherlands), offers a potential solution by reconstructing undersampled data using prior domain knowledge [11]. It has shown promising results across various tissue types and anatomical regions, aided by a training dataset that spans a wide range of contrasts, anatomical structures, and acceleration factors [12, 13, 14]. Despite its broad potential, the clinical utility of Adaptive CS-Net for non-contrast 3D whole-heart CMR using REACT has not been evaluated.

This study aimed to evaluate both the qualitative and quantitative image quality of six-fold undersampled 3D REACT images reconstructed using Adaptive CS-Net in healthy volunteers and patients with CHD. Serving as reference standards, conventional 3D bSSFP, and CS-reconstructed REACT images were included. To our knowledge, this is the first study to systematically compare Adaptive CS-Net with conventional CS reconstruction for REACT imaging in CHD, highlighting its potential as a high-quality, non-contrast-enhanced MRI solution.

## 2. Materials and Methods

### 2.1 Study Population

**Fig. 1.**
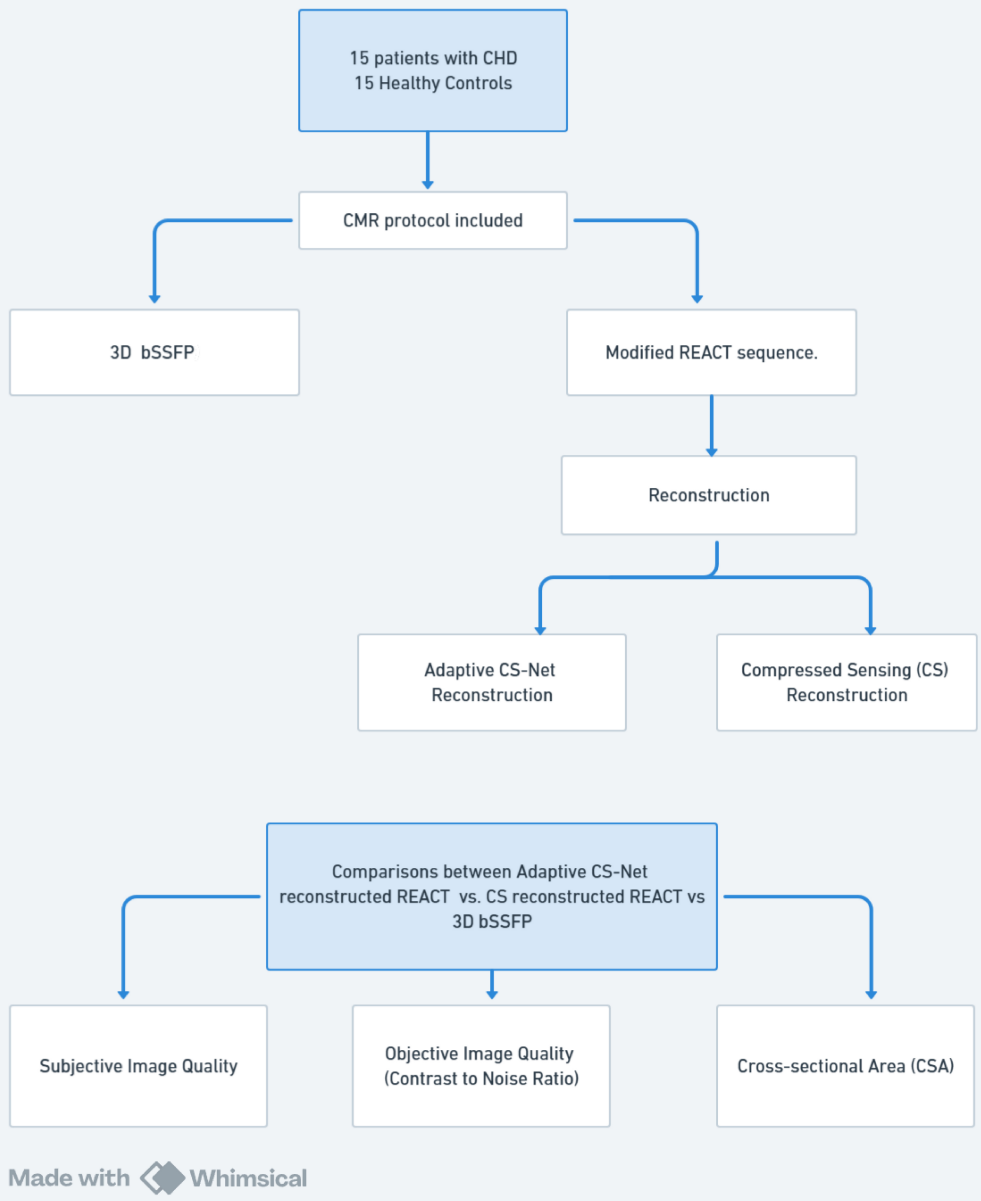
**Study design and analysis workflow**. CHD, congenital heart disease; CMR, cardiovascular magnetic resonance; REACT, relaxation-enhanced angiography without contrast and triggering; bSSFP, balanced steady-state free precession; 3D, three-dimensional.

30 participants, including 15 patients with CHD and 15 healthy volunteers, were prospectively recruited in the study. Fig. 1 presents a visual summary of the study design. A total of 30 participants were prospectively recruited for this study, including 15 patients with CHD and 15 healthy volunteers. Patients with CHD were included if they were scheduled to undergo clinically indicated non-contrast-enhanced CMR, allowing additional image acquisition without deviating from standard care. Healthy volunteers were recruited as a baseline comparison group; however, due to practical constraints, they were not age- or gender-matched to the patient group.

Exclusion criteria included patients scheduled for contrast-enhanced MRI and, within the CHD group, individuals with acquired or non-congenital forms of heart disease, to ensure focused evaluation of congenital cardiac anatomy.

The study included 30 participants (15 patients with CHD and 15 healthy controls) who underwent CMR imaging without contrast. The imaging protocol consisted of a 3D bSSFP sequence and a modified REACT sequence. The REACT data were reconstructed using both conventional CS and an Adaptive CS-Net algorithm. Image sets were then evaluated through subjective and objective quality assessments, including subjective image quality scores, contrast-to-noise ratio (CNR), and cross-sectional area (CSA) measurements. Comparative analyses were performed across all three imaging methods: Adaptive CS-Net REACT, CS REACT, and 3D bSSFP.

The local institutional review board approved the study. All patients and volunteers signed an informed consent form. A summary of patients’ characteristics can be found in Table 1.

**Table 1.** **Patient age, diagnosis, and procedures for participants with congenital heart disease**.

Patient number	Age (years)	Diagnosis	Procedures
1	52	Partial anomalous pulmonary venous drainage vein to left innominate vein	
2	15	Tetralogy of Fallot	Complete repair
3	5	Epstein anomaly	
4	14	Atrial septal defect	
5	17	Pulmonary stenosis, severe	
6	8	Tetralogy of Fallot	Complete repair
7	17	Cor triatriatum	
8	23	Tetralogy of Fallot	Complete repair, pulmonary valve replacement
9	16	Atrial septal defect	
10	7	Tetralogy of Fallot	
11	18	Ventricular septal defect (VSD)	VSD repair
12	27	AVCD, pulmonary atresia	Fontan procedure
13	12	Tetralogy of Fallot	Complete repair
14	0.5	AVCD	
15	17	VSD, aortic stenosis	Ross-Konno procedure

AVCD, atrioventricular canal defect.

### 2.2 MR Imaging Protocol

All scans were performed on a clinical cardiac dedicated 1.5 T MR system (Ingenia, Philips Healthcare, Best, Netherlands). For data acquisition, a 32-array dStream Torso coil (Philips HealthCare, Best, Netherlands) and the built-in spine coil elements were used. Automatic coil selection was employed.

The imaging protocol included a REACT sequence and a standard 3D bSSFP sequence for all cases of 3D whole-heart MRI without a contrast agent. A T2-prepulse and a group of inversion recovery prepulses were combined with an mDIXON technique for the REACT sequence. REACT imaging was based on a modified approach, including electrocardiogram (ECG) triggering in mid-diastole and respiratory navigator gating, which were added to the original REACT sequence. Six-fold undersampling was used for image acquisition to accelerate the REACT sequence. Other sequence parameters include echo time 1 (TE1)/ echo time 2 (TE2)/ repetition time (TR) = 1.85 ms (millisecond)/4.5 ms/6.8 ms, acquisition voxel size = 1.6 mm (millimeter) × 1.6 mm × 1.6 mm, reconstruction voxel size = 0.8 mm × 0.8 mm × 0.8 mm.

The single-phase 3D bSSFP sequence is respiratory-gated and ECG-triggered at end-systole with a spectral presaturation with inversion recovery fat saturation prepulse to null fat and a T2 prepulse to improve the myocardium to blood pool tissue contrast. For imaging acceleration of bSSFP, a SENSitivity Encoding (SENSE) factor of 1.5 on both anterior-posterior and foot-head directions was used. Other sequence parameters for the 3D bSSFP include TE/TR = 2.0 ms/4.0 ms, acquisition voxel size = 1.2 mm × 1.2 mm × 1.8 mm (foot-head × right-left × anterior-posterior), reconstruction voxel size = 0.98 mm × 0.98 mm × 0.9 mm.

### 2.3 MRI Reconstruction

The images acquired using the 3D bSSFP sequence were reconstructed in-line with the scanner. For the images acquired using the REACT sequence, the in-line CS reconstructions were saved for comparison, and the 6-fold undersampled raw k-space data were saved for off-line reconstruction using Adaptive CS-Net for comparison purposes. The following optimization problem was solved for Adaptive CS-Net reconstruction:

$$\min\,{||A\,x-y||}_{2}^{2}+\lambda_{1}\,{||\Phi \,x||}_{1}+\lambda_{2}\,{||R^{-1/2}\,x||}_{2}^{2},$$

Here, x is the target image, y is the measured k-space data, A includes coil sensitivities, Fourier transformation, and undersampling operator, Φ transforms the image data into wavelet space, and R represents coarse resolution data from the scanner, with λ1 and λ2 balancing data consistency and regularization.

Adaptive-CS-Net, which is based on the deep network form of iterative shrinkage-thresholding algorithm (ISTA-Net) [15], involves convolutional neural networks$F_{k}^{*}$ and F_k_ iteratively solving the optimization with an updating rule incorporating MRI priors for data consistency $e_{b,k}$, phase behavior $e_{\phi ,k}$, and background information $e_{b\,g,k}$:

$$x_{k}=x_{k-1}+F_{k}^{*}\,\left({\mathrm{soft}}_{\theta_{k}}\left(F_{k}\,\left(r_{k},e_{b,k},e_{\phi ,k},e_{b\,g,k}\right)\right)\right).$$

The networks were trained using a loss function that combines multiscale structural similarity and reconstruction error, aimed at preserving both structural integrity and contrast in the images. Rectified Adam optimizer is used for solving the optimization problem.

Adaptive-CS-Net was chosen in this work as the algorithm is maintained and supported by the MR vendor (Philips, Best, Netherlands). The neural networks in Adaptive-CS-Net were designed and trained by the MR vendor, leveraging their access to large-scale training datasets. The entire reconstruction framework was implemented in C, and the trained model was used for offline image reconstruction. The detailed implementation information can be found in [11, 12].

### 2.4 Image Analysis

Commercially available analysis software RadiAnt Dicom Viewer (2024.1, Medixant, Poznan, Poland) was used for image analysis and multi-planar reformatting. Coronary artery origins, cardiac chambers, and great vessels were analyzed for CNR and image quality.

### 2.5 Image Quality Visual Analysis 

Two pediatric cardiologists (FGG and MTH, each with over 15 years of experience in cardiovascular imaging) independently performed a blinded assessment of image quality, focusing on reformatted angiograms of each area of interest. All images were anonymized and randomly ordered before evaluation. Sequence labels, scan parameters, and other identifying information were removed to ensure blinding to the acquisition technique. Consensus grades were assigned following the independent reviews. Prior to scoring, both readers reviewed a set of representative cases and reached a consensus on how to apply McConnell’s criteria to ensure consistency in image quality assessment. According to the 5-point system that is described by McConnell *et al*. [16]; in grade 0: the structure was not visible; in grade 1: the structure was visible with markedly blurred borders; in grade 2: the structure was visible but with moderately blurred borders; in grade 3: the structure was visible with mildly blurred borders; in grade 4: the structure was visible with sharply defined borders.

### 2.6 Quantitative Image Quality Analysis

Quantitative image quality analysis was performed by calculating the CNR. For CNR calculation the following equation was used [17]:

$$C\,N\,R=\frac{S_{\text{blood }}-S_{\text{myo }}}{0.5\cdot \left(N_{\text{blood }}+N_{\text{myo }}\right)}$$

Here, S_blood_ and S_myo_ represent the mean signal intensity of blood in the region of interest and mean signal intensity from reference tissue (myocardium), and N_blood_ and N_myo_ stand for the noise of blood in the region of interest and noise from reference tissue (myocardium) respectively. The signal intensity of blood in the region of interest (S_blood_) and signal intensity from reference tissue (myocardium, S_myo_) were determined on identically reformatted images. Similarly, the noise was estimated from both these tissues respectively (N_blood_ and N_myo_).

Designated blood pool regions of interest were superior vena cava (SVC), right atrium (RA), right ventricle (RV), main pulmonary artery (MPA), left and right pulmonary artery (RPA and LPA, respectively), pulmonary veins (PVs), left atrium (LA), left ventricle (LV) and aorta.

### 2.7 Cross-Sectional Area Measurements

CSA measurements of the transverse aortic arch, right ventricular outflow tract (RVOT) at the valvar and supravalvar levels, the MPA before its bifurcation, proximal segments of the RPA and LPA, proximal transverse aortic arch, and aortic isthmus, were carefully obtained at closely matched locations within the vessels. These measurements were then compared across three distinct sets of images to assess the consistency of the observations within the same evaluator.

### 2.8 Statistical Analysis

SPSS (version 29.02, IBM, Armonk, NY, USA) and Python 3.12.7 (packaged by Anaconda, Inc., Austin, TX, USA) were used for statistical analysis. The Shapiro-Wilk test was applied to evaluate the normality of the CSA measurements and CNR values. A paired *t*-test and Wilcoxon signed-rank test were performed for normally and non-normal distributed data, respectively. The Shapiro-Wilk test was conducted on the differences in CSA measurements to validate the assumptions of the Bland-Altman analysis, which was used to assess the consistency between imaging techniques. The nonparametric Friedman test and the Dunn test were used for multiple group image quality comparison between the three sets of images. To evaluate whether our sample size provided adequate statistical power, we performed a post hoc power analysis using Kendall’s W values derived from the Friedman test. For five vessels (right lower PV (RLPV), right upper PV (RUPV), LA, ascending aorta (AA), SVC), the effect sizes were large (W ≥ 0.2), corresponding to a power exceeding 90% at a significance level of 0.05. The remaining vessels showed moderate effect sizes (0.1 ≤ W < 0.2), which still supported reasonable power levels for within-subject comparisons. These findings indicate that our sample of 30 patients was sufficient to detect clinically meaningful differences across the three imaging modalities.

## 3. Results

ECG triggered and respiratory navigator gated REACT and 3D bSSFP acquisitions and reconstructions were completed successfully in all healthy volunteers and patients with CHD (n = 30). The mean age was 16.5 (7 female, 8 male) for patients with CHD and 19.2 (13 female, 2 male) for healthy volunteers. All participants were in sinus rhythm. Two patients (mean age: 4.2) underwent imaging under general anesthesia. Table 1 shows the patient cohort with corresponding CHD diagnoses. The REACT sequence has an average acquisition time of 5.3 minutes while the 3D bSSFP sequence averages 4.2 minutes. Although the mDIXON technique typically requires long acquisition time due to its multi-echo approach, applying a 6-fold undersampling factor reduced the total acquisition time to a level comparable to the 3D bSSFP sequence. Average reconstruction times are 43 seconds for CS and 28 seconds for Adaptive-CS-Net (*p* < 0.05). The Adaptive CS-Net reconstruction is done on a clinical workstation equipped with a NVIDIA A6000 GPU.

### 3.1 Image Quality

Image quality scores of predefined areas of interest are compared between Adaptive CS-Net reconstructed, CS reconstructed, and 3D bSSFP images and presented in Table 2.

**Table 2.** **Image quality score comparison between Adaptive CS-Net reconstructed REACT, CS reconstructed REACT, and 3D bSSFP**.

	Median image quality score			
	Adaptive CS-Net REACT	CS-REACT	3D bSSFP	Equality test *p*-value
Superior vena cava	3^‡,†^ (3, 4)	3 (3, 3)	3 (2, 3)	<0.001
Right atrium	3^‡^ (3, 3)	3 (2, 3)	3* (3, 4)	<0.001
Tricuspid valve	0 (0, 1)	0 (0, 1)	1 (0, 1)	0.018
Right ventricle	3^‡^ (3, 3)	2 (2, 3)	4* (3, 4)	<0.001
Main pulmonary artery	3^‡^ (3, 4)	3 (2, 3)	4* (3, 4)	<0.001
Right pulmonary artery	3 (3, 4)	3 (3, 3)	3* (3, 4)	<0.001
Left pulmonary artery	3 (3, 4)	3 (3, 3)	3* (3, 4)	<0.001
Left pulmonary veins	3^†^ (2.25, 4)	3* (2, 3)	1 (1, 2)	<0.001
Right pulmonary veins	3^†^ (2, 3.75	3* (2, 3)	1 (1, 2)	<0.001
Left atrium	3^‡^ (2.25, 3)	2.5 (2, 3)	3* (2.25, 3)	<0.001
Mitral valve	1 (0, 2)	1 (0, 2)	1 (0, 1)	0.069
Left ventricle	3^‡^ (2.25, 3)	2 (2, 3)	3.5^†,^* (3, 4)	<0.001
Aortic valve	1 (0, 1)	1 (0, 1)	0.5 (0, 1)	0.532
Ascending aorta	3^‡^ (3, 4)	3 (3, 3)	3.5* (3, 4)	0.002
Left coronary artery	3 (2, 3)	2 (2, 3)	3* (2.25, 3)	<0.001
Right coronary artery	2 (1, 3)	2 (1, 3)	3* (2, 3)	0.011
Upper thoracic and neck veins	4^†^ (4, 4)	4* (3, 4)	2 (1, 3)	<0.001
Upper thoracic and neck arteries	4^†^ (4, 4)	4* (3, 4)	2 (1.5, 2)	<0.001

Columns 2, 3, and 4 give the median image quality scores and the 25% and 75% percentiles in parentheses. The *p*-values of pairwise comparisons of image quality scores performed by Friedman and Dunn’s test are given with symbols as follows ^‡^: *p* < 0.05 H_0_: Adaptive CS-Net REACT = CS REACT, ^†^: *p* < 0.05, H_0_: Adaptive CS-Net REACT = 3D bSSFP, *: *p* < 0.05 H_0_: CS-REACT = 3D bSSFP. Column 5 (the last column) gives the results of the Friedman test for the equality of all image quality scores across 3 methods. Bonferroni corrections are not applied to the *p*-values. 3D bSSFP, three-dimensional balanced steady state free precession; CS, compressed sense; REACT, relaxation-enhanced angiography without contrast and triggering.

The Adaptive CS-Net reconstructed REACT provided superior or comparable image quality for all regions of interest compared to CS reconstructed REACT and 3D bSSFP images. Furthermore, the Adaptive CS-Net and CS reconstructed images demonstrated significantly superior image quality for both left and right PVs compared to standard 3D bSSFP sequence, with all *p*-values < 0.001. For PVs, the median image quality scores between these CS and Adaptive CS-Net reconstructed images were similar (left PVs: Adaptive CS-Net REACT: 3 (2.25 to 4), CS reconstructed REACT: 3 (2 to 3), *p* = 0.288; right PVs: Adaptive CS-Net REACT: 3 (2 to 3.75), CS reconstructed REACT: 3 (2 to 3), *p* = 0.417).

Additionally, both the Adaptive CS-Net and CS reconstructed REACT provided enhanced image quality for upper thoracic and neck vessels compared to 3D bSSFP, for upper thoracic and neck arteries, *p*-value < 0.001, for upper thoracic and neck veins, *p*-value < 0.001.

The Adaptive CS-Net reconstructed REACT offered superior image quality compared to the CS reconstructed REACT for all cardiac chambers and great vessels, including SVC, ascending aorta (AAo), and MPA. Additionally, the 3D bSSFP sequence provided statistically significantly better image quality for the AAo than the CS reconstructed REACT images.

The 3D bSSFP sequence provided superior image quality for intracardiac structures compared to the CS reconstructed REACT sequence for the left and right coronary artery origins, all cardiac chambers, and pulmonary arteries. However, compared to the Adaptive CS-Net reconstructed REACT, the 3D bSSFP sequence only demonstrated better image quality for the LV.

**Fig. 2.**
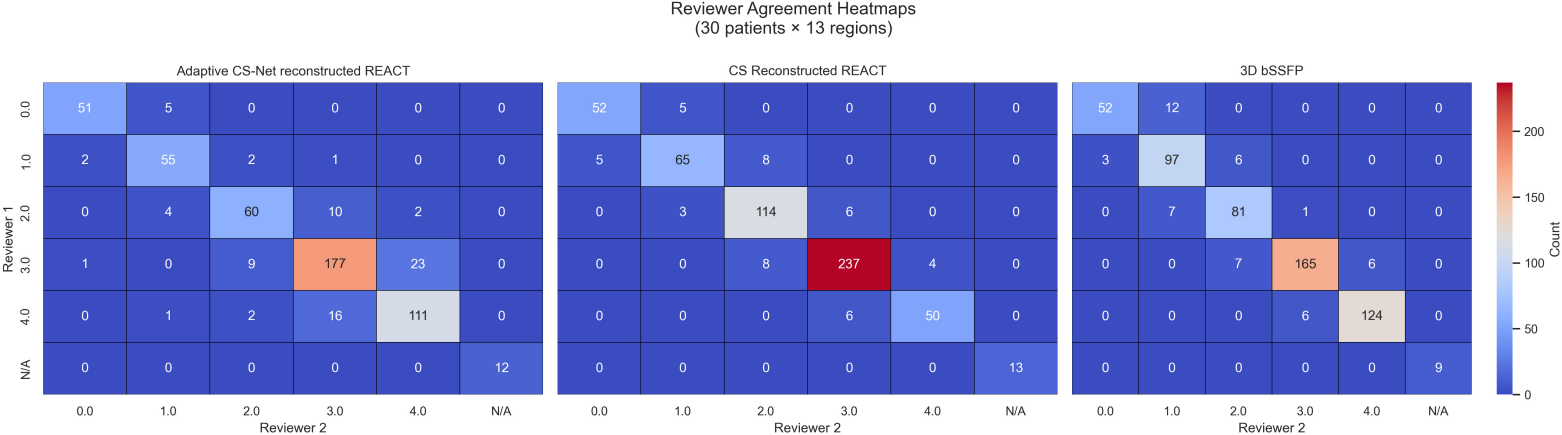
**Reviewer agreement heatmaps based on Cohen’s kappa analysis for interrater reliability across three imaging modalities**. These heatmaps visualize interrater agreement between two reviewers for the qualitative assessment of cardiac image quality across three MRI reconstruction techniques: Adaptive CS-Net reconstructed REACT (left), CS reconstructed REACT (center), and 3D bSSFP (right). Each matrix cell represents the count of ratings given by Reviewer 1 (y-axis) and Reviewer 2 (x-axis), for image quality scores ranging from 0 to 4. Higher diagonal counts indicate stronger agreement. Cohen’s kappa values were computed for each modality to assess the strength of interrater agreement, with red hues denoting higher agreement counts. 3D bSSFP, three-dimensional balanced steady state free precession; CS, compressed sense; REACT, relaxation-enhanced angiography without contrast and triggering; MRI, magnetic resonance imaging.

A Weighted Cohen’s κ test was computed across all rated structures for each modality to quantify interrater agreement Fig. 2. The resulting κ coefficients were 0.93 for Adaptive CS-Net reconstructed REACT, 0.97 for CS reconstructed REACT, and 0.98 for 3D bSSFP—values that all fall into the “almost perfect” agreement between readers for each imaging modality [18].

### 3.2 CNR Measurements

The CNR results are summarized in Table 3, with the rightmost column reporting the Friedman *p*-value for the global test of equal medians across Adaptive CS-Net reconstructed REACT, CS reconstructed REACT, and 3D bSSFP. Significant overall differences (*p* < 0.05) were observed in nine of the thirteen regions (right atrium, right ventricle, all four pulmonary veins, left atrium, ascending aorta, and superior vena cava). Four regions—the main pulmonary artery, left pulmonary artery, right pulmonary artery, and left ventricle—showed no significant modality effect (*p* ≥ 0.05), although Adaptive CS-Net reconstructed REACT had the numerically highest median in each of these.

**Table 3.** **Median contrast to noise ratio comparison across imaging methods**.

Regions of interest	Adaptive CS-Net-REACT	CS-REACT	3D bSSFP	Friedman *p-*value
Right atrium	15.04^†^	12.53	10.9	0.012
Right ventricle	14.12	10.84	14.54*	0.048
Main pulmonary artery	12.46	10.47	12.24	0.289
Left pulmonary artery	11.26	9.54	10.26	0.131
Right pulmonary artery	11.81	10.19	9.34	0.497
Left upper pulmonary vein	5.40^†^	5.53*	0.97	0.007
Left lower pulmonary vein	6.33^†^	5.84*	2.27	0.006
Right upper pulmonary vein	5.49^†^	6.74*	1.18	0.000
Right lower pulmonary vein	6.71^†^	6.41*	1.26	0.002
Left atrium	16.80^†^	14.60*	8.29	0.000
Left ventricle	16.77	13.87	13.88	0.202
Ascending aorta	16.44^‡,†^	13.61*	9.8	0.000
Superior vena cava	12.59^†^	11.02*	7.62	0.000

Values are the median CNRs obtained from 30 patients for each vessel using Adaptive CS-Net reconstructed REACT, compressed-sensing REACT (CS), and 3D bSSFP. Superscripts mark statistically significant pairwise differences (Friedman → Dunn, α = 0.05): ^‡^: *p* < 0.05 H_0_: Adaptive CS-Net REACT = CS REACT, ^†^: *p* < 0.05 H_0_: Adaptive CS-Net REACT = 3D bSSFP, *: *p* < 0.05 H_0_: CS REACT = 3D bSSFP. The last column (equality test *p*-value) shows the *p*-value of the equality of all measurements (H_0_: Adaptive CS-Net REACT = CS REACT = 3D bSSFP). 3D bSSFP, three-dimensional balanced steady state free precession; CS, compressed sensing; REACT, relaxation-enhanced angiography without contrast and triggering; CNR, contrast-to-noise ratio.

In post-hoc Dunn tests, Adaptive CS-Net reconstructed REACT significantly outperformed 3D bSSFP in the right atrium (15.04 vs. 10.90), four pulmonary veins, left atrium (16.80 vs. 8.29), superior vena cava (12.59 vs. 7.62), and ascending aorta (16.44 vs. 9.80) where it also surpassed CS reconstructed REACT (16.44 vs. 13.61), (all *p-*values < 0.05). Similarly, CS reconstructed REACT yielded significantly higher CNR than 3D bSSFP in the four pulmonary veins, left atrium, ascending aorta, and superior vena cava (all *p* ≤ 0.05), whereas the right ventricle was the only region of interest in which 3D bSSFP outperformed CS reconstructed REACT (median CNR: 14.54 vs. 10.84).

### 3.3 Cross-Sectional Area Measurements

The results of CSA measurements are shown in CSA measurements showed high consistency, with no significant differences between methods. Reliability was excellent across methods: the intraclass correlation coefficient ICC(2,1) values for vessel-specific CSA measurements ranged from 0.93 to 0.98, confirming that 3D bSSFP, CS reconstructed REACT, and Adaptive CS-Net reconstructed REACT provide highly consistent results despite the wider Bland-Altman limits of agreement.

**Fig. 3.**
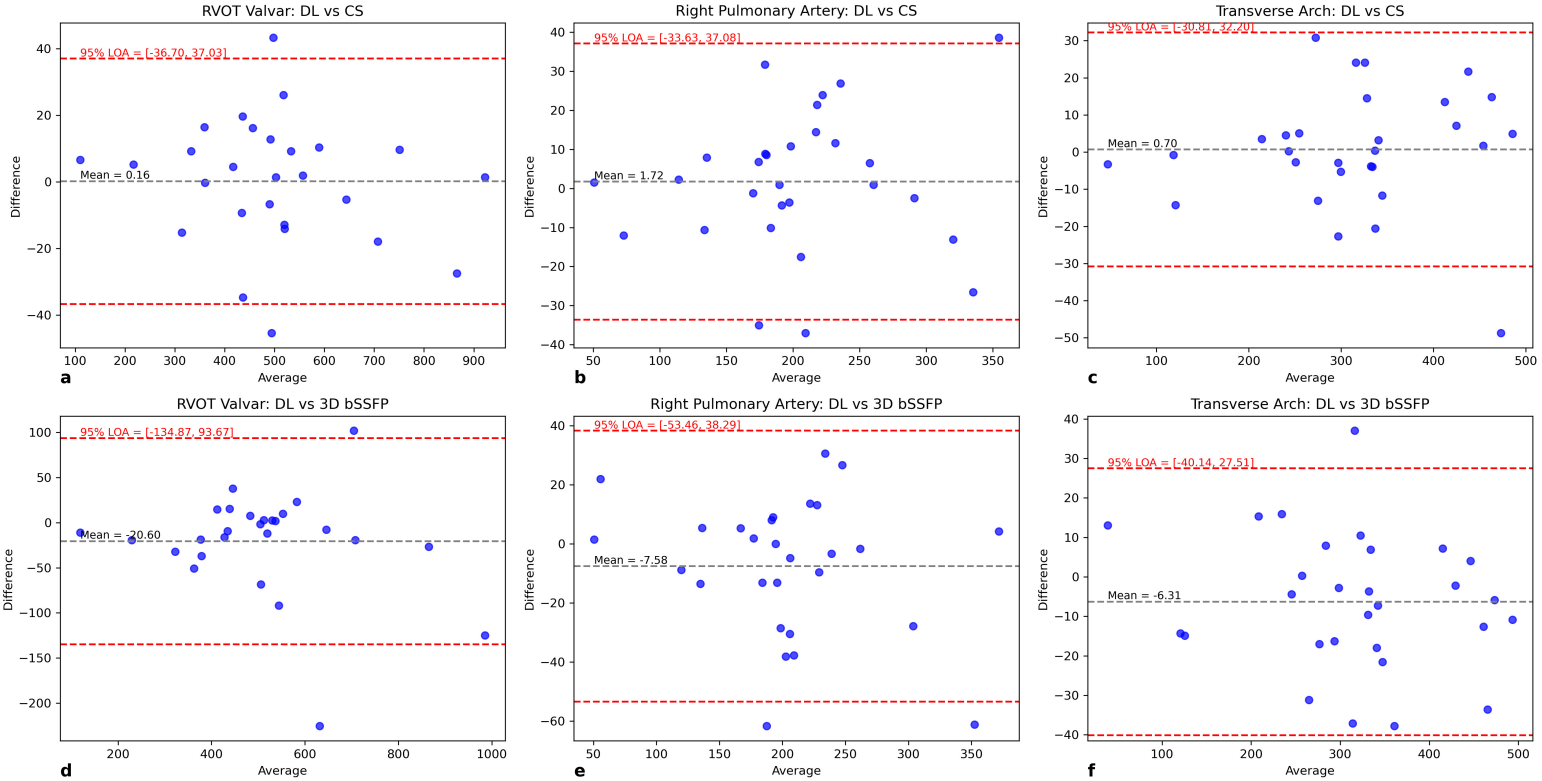
**Bland-Altman analysis of cross-sectional area comparisons between imaging techniques**. The Blant-Altman analysis comparing cross-sectional areas (CSA) (millimeter square (mm^2^)) between 6-fold accelerated acquisition with deep learning (DL) (Adaptive CS-Net) reconstructed REACT (relaxation-enhanced angiography without contrast and triggering) and 6-fold accelerated acquisition with conventional CS (compressed sensing) reconstructed REACT (a–c) and between 6-fold accelerated acquisition with Adaptive CS-Net reconstructed REACT and 3D bSSFP (d–f). The black line indicates the mean difference of the diameter measurements, whereas the red lines represent the 95% confidence interval. The bias reflects the *p*-value of the regression coefficient when the mean difference is regressed against the average. (a) Coaxial right ventricular outflow tract (RVOT) at valvar level CSA measurements of Adaptive CS-Net and CS reconstructed REACT images demonstrate excellent agreement with a mean difference of 0.16 mm^2^ (95% confidence interval (CI) –36.70 to 37.03). (b) Coaxial right pulmonary artery (RPA) CSA measurements of Adaptive CS-Net and CS reconstructed REACT images demonstrate excellent agreement of a mean difference of 1.72 mm^2^ (95% CI –33.63 to 37.08). (c) Coaxial Transverse Arch CSA measurements of Adaptive CS-Net and CS reconstructed REACT demonstrate excellent agreement of a mean difference of 0.7 mm^2^ (95% CI –30.81 to 32.20). (d) Coaxial RVOT at valvar level CSA measurements of Adaptive CS-Net and 3D bSSFP images demonstrate good agreement with a mean difference of –20.6 mm^2^ (95% CI –134.87 to 93.67). (e) Coaxial RPA CSA measurements of Adaptive CS-Net and 3D bSSFP images demonstrate excellent agreement of a mean difference of –7.58 mm^2^ (95% CI –53.46 to 38.29). (f) Coaxial Transverse Arch CSA measurements of Adaptive CS-Net and 3D bSSFP demonstrate excellent agreement of a mean difference of –6.31 mm^2^ (95% CI –40.14 to 27.51). 3D bSSFP, three-dimensional balanced steady state free precession.

Table 4 and Fig. 3 CSA measurements showed high consistency, with no significant differences between methods. Reliability was excellent across methods: ICC(2,1) values for vessel-specific CSA measurements ranged from 0.93 to 0.98, confirming that 3D bSSFP, CS reconstructed REACT, and Adaptive CS-Net reconstructed REACT provide highly consistent results despite the wider Bland-Altman limits of agreement.

**Table 4.** **Comparison of cross-sectional area measurements (millimeter square) between Adaptive CS-Net reconstructed REACT, CS reconstructed REACT and 3D bSSFP**.

	CS reconstructed REACT	3D bSSFP	Adaptive CS-Net reconstructed REACT	*p*-value
RVOT valvar level	499.1 ± 178.1	519.9 ± 186.6	499.3 ± 174.9	0.892
RVOT supravalvar	434.5 ± 159.2	467.7 ± 171.2	436.9 ± 163.0	0.727
MPA pre bifurcation	492.9 ± 174.1	533.7 ± 192.1	481.3 ± 167.1	0.584
LPA proximal	274.4 ± 108.0	298.6 ± 133.9	274.4 ± 124.6	0.788
LPA distal	229.4 ± 89.9	255.7 ± 115.1	232.7 ± 103.5	0.630
RPA proximal	240.1 ± 74.0	251.5 ± 87.6	243.0 ± 75.2	0.745
RPA distal	208.0 ± 66.2	218.3 ± 89.2	208.4 ± 67.7	0.684
Isthmus aorta	235.7 ± 71.9	242.3 ± 6.7	237.9 ± 75.7	0.944
Transverse arch	312.5 ± 106.1	319.5 ± 110.2	304.1 ± 107.0	0.967

The numbers in the chart represent the average cross-sectional area measurements along with their standard deviations. The last column displays the *p*-values from the test that hypothesizes the measurements are identical across all methods. The results indicate that the measurements are similar across all methods. 3D bSSFP, three-dimensional balanced steady state free precession; CS, compressed sense; LPA, left pulmonary artery; MPA, main pulmonary artery; REACT, relaxation-enhanced angiography without contrast and triggering; RPA, right pulmonary artery; RVOT, right ventricular outflow tract.

### 3.4 Visual Comparison of Imaging Techniques

**Fig. 4.**
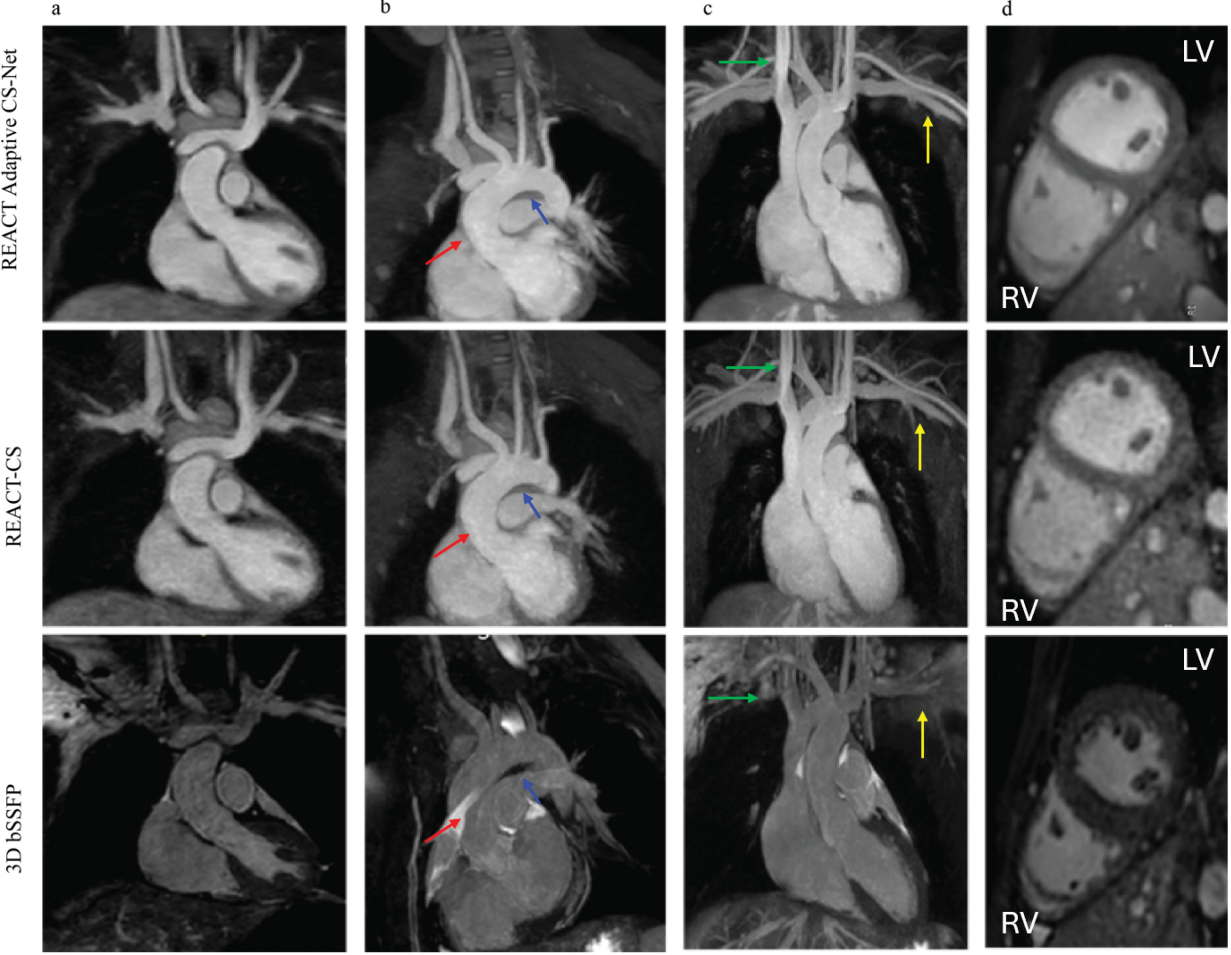
**Comparison of thoracic vessel imaging using Adaptive CS-Net reconstructed REACT, CS reconstructed REACT, and 3D bSSFP**. The upper thoracic and neck vessels in the coronal view (column a) and multiplanar reformats (columns b and c) of the ascending aorta (AAo, red arrow) and aortic arch (blue arrow) are displayed. The REACT (relaxation-enhanced angiography without contrast and triggering) technique produced uniform signals for the AAo and aortic arch, offering better border delineation compared to 3D bSSFP, which was maintained in both Adaptive CS-Net and CS (compressed sense) reconstructed images. Additionally, both REACT techniques provided higher-resolution images of the upper thoracic (yellow arrow) and neck vessels (green arrow) compared to 3D bSSFP. Multiplanar reformats of the right and left ventricles in the sagittal view (column d) show mild blurring in CS reconstructed REACT images which were reduced with Adaptive CS-Net reconstruction. The left ventricular endocardial border, papillary muscles, and right ventricular trabeculations and papillary muscles were sharply defined with 3D bSSFP compared to REACT techniques. Comparing the Adaptive CS-Net reconstructed REACT and CS reconstructed REACT, we can see significant noise reduction in the images, illustrating better image quality from Adaptive CS-Net reconstructed REACT. LV, left ventricle; RV, right ventricle; 3D bSSFP, three-dimensional balanced steady state free precession.

The 6-fold undersampled REACT data reconstructed with the Adaptive CS-Net technique, compared to CS reconstruction and 3D bSSFP sequence, are presented in Fig. 4. Uniform windowing levels are consistently applied across all images, including those depicted in Fig. 4 and other figures presented in this work. The Adaptive CS-Net reconstruction demonstrated reduced noise levels and improved image quality compared to CS reconstruction for all cardiac chambers. Also, both the Adaptive CS-Net and CS-reconstructed images delivered enhanced image quality for upper thoracic and neck vessels compared to 3D bSSFP.

**Fig. 5.**
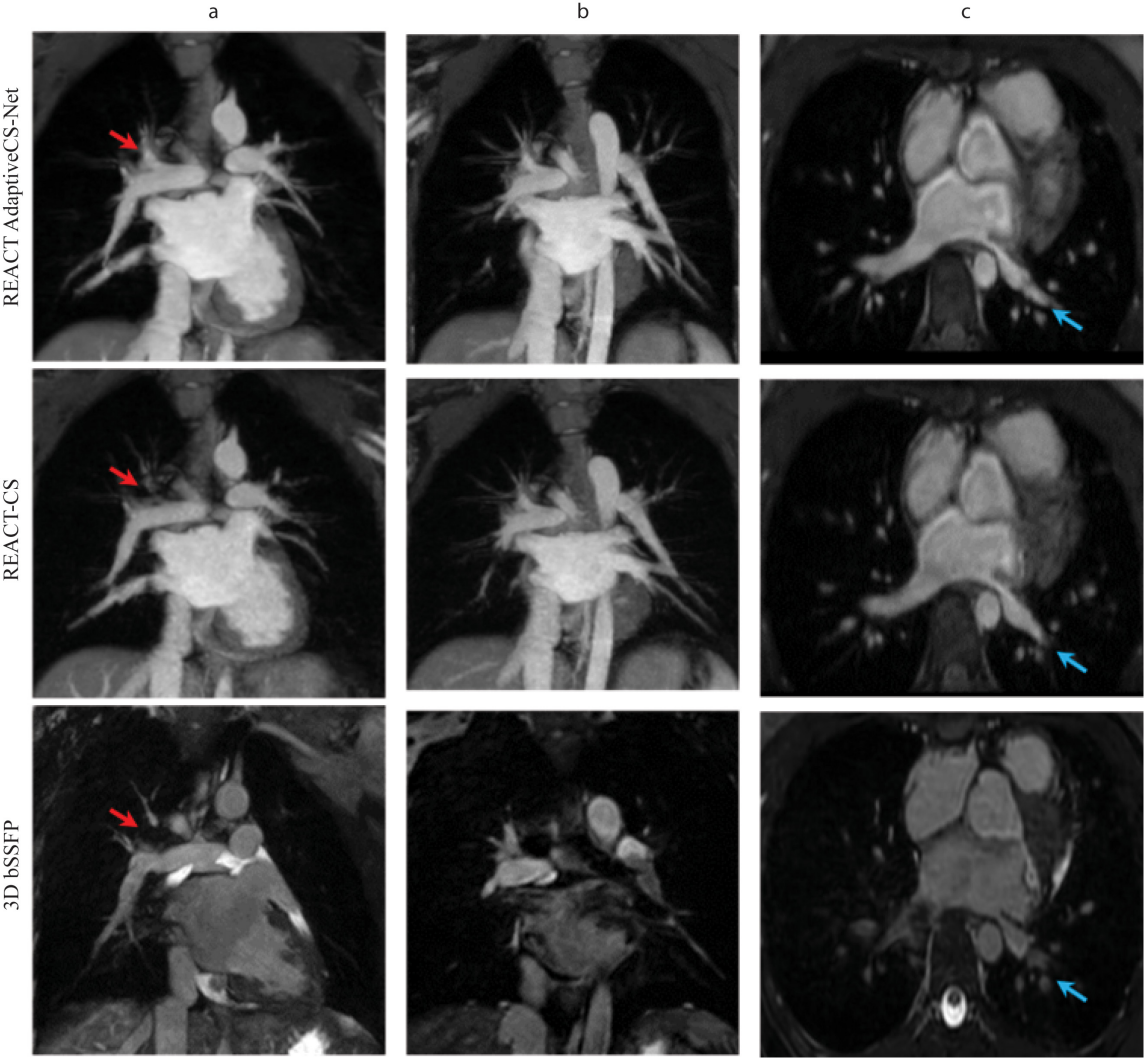
**Performance comparisons of 3D whole-heart techniques in demonstrating pulmonary veins and pulmonary arteries**. Acquisitions were performed with relaxation-enhanced angiography without contrast and triggering (REACT) and 3D bSSFP. REACT images were reconstructed with Adaptive CS-Net and compressed sensing (CS). Multiplanar reconstructions of pulmonary veins with close-up views show that REACT techniques provided significantly better image quality by suppressing flow and off-resonance artifacts. In column a, red arrows represent the upper branch of the right pulmonary artery, which was visualized with Adaptive CS-Net reconstructed REACT but not with CS reconstructed REACT and 3D bSSFP. Adaptive CS-Net reconstruction was not only a useful method in imaging pulmonary veins but also proved to be a significant aid in imaging pulmonary arteries. Column b shows left pulmonary veins and right upper pulmonary vein. Signal loss seen with 3Db SSFP is not seen with REACT images, significantly improving image quality. In column c, the blue arrows represent the left lower pulmonary veins. REACT images demonstrate the left lower pulmonary veins in full length and better resolution, while 3D bSSFP exhibited dephasing artifacts and reduced image quality. 3D bSSFP, three-dimensional balanced steady state free precession.

Fig. 5 demonstrated the attenuation of flow-related and off-resonance artifacts in PVs with Adaptive CS-Net and CS reconstructed REACT images compared to 3D bSSFP imaging. In Fig. 3, image quality was significantly better in Adaptive CS-Net and CS reconstructed images compared to 3D bSSFP images. Still, Adaptive CS-Net and CS reconstructed images showed similar image quality in the left and right PVs.

**Fig. 6.**
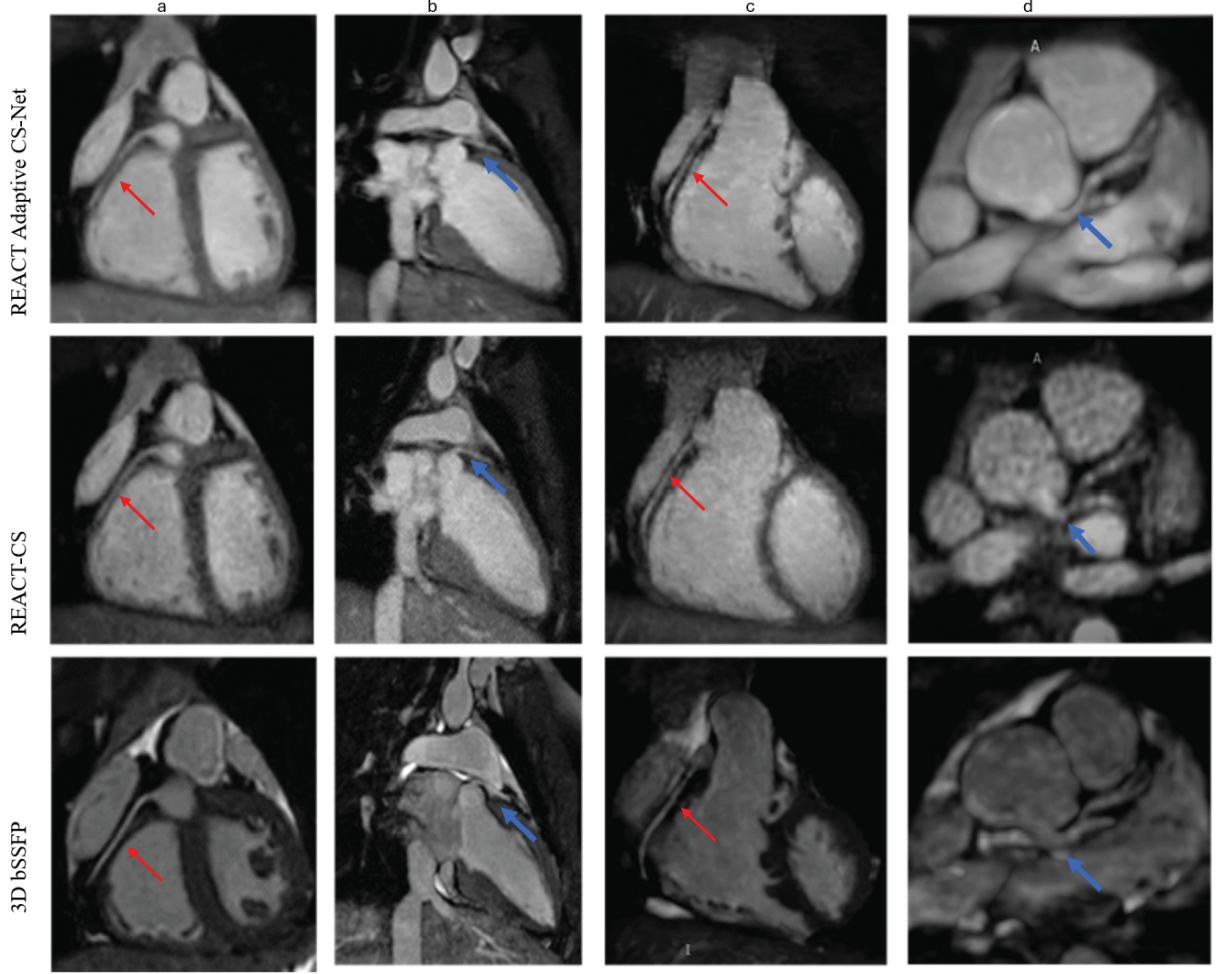
**Coronary artery imaging: adaptive CS-Net vs. CS reconstructed REACT and 3D bSSFP**. All methods successfully visualized the coronary arteries. Multiplanar reformats (with the right coronary artery labeled by a red arrow in columns a and c, and the left main coronary artery labeled by a blue arrow in columns b and d) demonstrated that 3D bSSFP provided superior image quality compared to relaxation-enhanced angiography without contrast and triggering (REACT) images reconstructed with conventional compressed sensing (CS), which showed blurred vessel borders. In contrast, Adaptive CS-Net reconstruction improved image sharpness, providing higher signal quality, reduced noise, and less blurring than standard CS reconstruction. 3D bSSFP, three-dimensional balanced steady state free precession.

The 3D bSSFP imaging outperformed the CS reconstructed REACT for imaging intracardiac morphology, including the origin of the coronary arteries, pulmonary arteries, and cardiac chambers, except for the LA (Fig. 6). But the Adaptive CS-Net reconstruction improved the image quality compared to CS reconstruction.

## 4. Discussion

This study offers valuable insights into advancing non-contrast-enhanced CMR for CHD patients by integrating Adaptive CS-Net with the REACT sequence. First, DL-based reconstruction with the REACT sequence enhances image quality. Adaptive CS-Net consistently produced high-resolution, high-contrast images that matched or surpassed the quality of conventional CS reconstructed REACT images and 3D bSSFP sequences, which served as the reference standard. The algorithm’s high CNR and quality scores underscore its diagnostic potential, particularly valuable in CHD cases where non-contrast imaging reduces risks and costs for patients requiring lifelong monitoring. This approach generated stable, high-quality “bright blood” images essential for assessing complex thoracic anatomy without contrast agents.

In addition to enhancing image quality, the DL-based reconstruction with the REACT sequence improves workflow efficiency. With six-fold acceleration, REACT images were captured in an average of 5.3 minutes, improving scan time predictability and clinical workflow. Reconstruction times were also notably faster, with Adaptive CS-Net completing reconstructions in just 28 seconds, demonstrating its feasibility for routine clinical use. By overcoming the limitations of bSSFP sequences and traditional CS reconstructions, this study positions REACT with DL-based reconstruction as a promising, patient-centered solution for high-resolution, non-contrast CMR in CHD, providing both enhanced imaging and workflow efficiency.

Adaptive CS-Net uses an iterative, DL-based reconstruction scheme inspired by CS theory, refining reconstruction assumptions through deep neural networks trained on large datasets [11] using fully sampled data. Unlike CS, which requires iterative reconstruction for each subject [19]. Adaptive CS-Net leverages prior knowledge to both reduce computational time and improve image quality, particularly in terms of noise reduction. While CS has proven beneficial in reducing REACT scan times, it has limitations at higher acceleration levels, which can degrade image quality [20]. Adaptive CS-Net addresses these challenges by providing fast and accurate reconstructions. In the rapidly advancing field of medical imaging, the adoption of deep learning models, particularly those based on transformer architectures, has been explored for a variety of applications. While transformers have shown remarkable success in areas like natural language processing and computer vision, their application in MRI reconstruction raises several practical concerns. Transformers are inherently resource-intensive, requiring significant computational power and memory. This is particularly challenging in MRI reconstruction, where the input data are large and high-dimensional. The self-attention mechanism in transformers scales quadratically with the input size, making them less efficient for the large-scale data typically involved in MRI images. This demand can be prohibitive, especially in clinical settings that may not have access to high-performance computing resources. Therefore, transformer-based models are not ideally suitable for our setting.

Recent studies have demonstrated the clinical efficacy of Adaptive CS-Net across various MRI applications. Pednekar *et al*. [21], for example, found that Adaptive CS-Net enabled high k-space undersampling in 2D cine images, maintaining volumetric and functional indices comparable to fully sampled k-space data while reducing breath-hold times by 57%. Another study highlighted the diagnostic strength of Adaptive CS-Net in non-contrast coronary magnetic resonance angiography (MRA), achieving high image quality and comparable diagnostic performance to coronary tomographic angiography within a feasible clinical timeframe, offering a safe alternative without contrast media risks [15]. Furthermore, the workflow has already been integrated into the new Philips scanner with GPU embedded on the workstation. So the Adapative CS-Net can be readily used in clinical practice.

Building on these advancements, our study found that Adaptive CS-Net reconstruction provides superior image quality and higher CNR for essential structures like the AAo and SVC compared to CS reconstruction and 3D bSSFP. Enhanced visualization of these vessels is critical for detecting vascular abnormalities and guiding clinical decisions, especially in CHD and other complex cardiovascular conditions. By delivering clearer, more detailed images without contrast agents, Adaptive CS-Net can improve diagnostic confidence and reduce the need for repeat scans, enhancing both patient outcomes and cardiac MRI efficiency. Additionally, Adaptive CS-Net and CS reconstructed REACT images demonstrated enhanced quality for upper thoracic and neck vessels, including arteries and veins, over 3D bSSFP. The REACT sequence captures arterial and venous structures with high spatial resolution, and its integration with Dixon and T2 preparation pulses in the REACT sequence, rather than T2-prepared bSSFP alone, has proven effective in vascular imaging, even in regions with fast-flowing blood [22]. Isaak *et al*. [23] found that REACT enables contrast-free and reliable imaging of the entire thoracic vasculature with CHD while providing higher image quality compared to the commonly used first-pass-contrast enhanced magnetic resonance angiography (CMRA) and similar image quality compared to high-resolution contrast-enhanced steady-state CMRA. Our findings support these results, showing significant quality improvements for upper thoracic and neck vessels compared to 3D bSSFP in REACT sequences. Nonetheless, 3D bSSFP remains superior or comparable in depicting specific intracardiac structures, such as cardiac chambers, coronary arteries, and atrioventricular valves.

CMR is considered the gold standard for assessing anomalous connections and stenosis of the pulmonary veins [24]. CMR is particularly effective in detecting anomalous connections and stenoses within the pulmonary veins due to its ability to capture detailed anatomical information. Notably, bSSFP sequences yield a high signal-to-noise ratio and improved contrast between blood and surrounding tissues without the need for contrast agents. However, bSSFP may produce signal voids in non-contrast 3D acquisitions of pulmonary veins. This issue arises from the proximity of pulmonary veins to the lungs, which causes significant resonance frequency shifts that can lead to severe signal void or banding artifacts, especially in the presence of off-resonance blood flow. Blood flow in the presence of off-resonance is also a potential source of artifacts in steady-state free precession (SSFP) imaging [25]. Conversely, REACT utilizes a modified DIXON technique, offers reduced sensitivity to inhomogeneities in the magnetic field, and provides robust fat and background suppression. Therefore, it provides high-resolution scans in the large field of views and allows application in higher magnetic fields such as 3 Tesla, where inhomogeneities are expected to be higher [26]. Pennig *et al*. [27] demonstrated that the 3D-modified REACT sequence outperforms four-dimensional contrast-enhanced magnetic resonance angiography (CEMRA) in terms of image quality when imaging the pulmonary vasculature in CHD patients, providing more robust and reliable results. This study aligns with these findings, showing that Adaptive CS-Net and CS reconstructed REACT significantly enhance image quality of pulmonary veins compared to the 3D bSSFP sequence. This improvement is crucial for CHD patients, as clearer and more precise imaging contributes to better diagnosis and management of these complex cases.

Finally, the 3D bSSFP sequence demonstrated superior image quality scores compared to CS-reconstructed REACT images for both coronary artery and cardiac chamber visualization. In pediatric patients, traditional 3D whole-heart (WH) imaging typically utilizes the systolic rest period for coronary artery imaging, as it is often longer than the diastolic rest period. In this study, REACT images were acquired during mid-diastole, whereas the bSSFP sequence was acquired at end-systole. The superior coronary artery image quality observed with the 3D bSSFP sequence may be attributed to reduced cardiac motion during the longer systolic rest period, while REACT images exhibited mild blurring, likely due to more intracavitary motion associated with rapid ventricular filling during the diastolic rest period. Additionally, 3D bSSFP yielded higher image quality scores for cardiac chambers compared to CS-reconstructed REACT images. This finding is consistent with prior work by Hussain *et al*. [17], who reported that systolic imaging provides improved visualization of cardiac chambers during 3D whole-heart imaging, regardless of heart rate, likely due to more favorable blood exchange dynamics during this phase compared to diastolic imaging.

Our findings highlight the complementary strengths of REACT and 3D bSSFP sequences. While REACT with Adaptive CS-Net reconstruction excels in vascular imaging, particularly in pulmonary veins, the upper thoracic vasculature, and great vessels, 3D bSSFP remains advantageous for detailed visualization of intracardiac structures such as coronary arteries and cardiac chambers. Given this, a hybrid imaging protocol combining REACT and 3D bSSFP may offer an optimal solution for comprehensive CMR assessment in CHD patients.

This study has several limitations. As a single-center investigation with a relatively small and age-diverse cohort, the generalizability of our findings to broader clinical settings or MRI systems from other vendors may be limited. Future multicenter studies with larger, more diverse populations are warranted to validate and extend these results. Although subjective image quality assessments were performed in a blinded manner, the distinct visual characteristics of each sequence may have inadvertently introduced reviewer bias. Furthermore, the heterogeneity of CHD diagnoses may have contributed to variability in the imaging outcomes. While this diagnostic diversity reflects the complexity of real-world clinical practice, we acknowledge that it may influence the consistency and interpretation of the results. One point of limitations involves the selection of reference tissue for CNR calculation. In our study, myocardium was selected as the reference tissue due to its clinical relevance, consistency, and reliability. The REACT sequence is primarily used to evaluate cardiovascular anatomy, where blood-myocardium contrast is often the most diagnostically important. This choice aligns with the clinical focus of the sequence. Moreover, myocardial signal tends to be stable and less susceptible to inter-patient variability compared to fat or skeletal muscle, which may be inconsistently suppressed or exhibit signal inhomogeneities. Nonetheless, we acknowledge that while myocardium is a commonly used reference tissue in traditional CNR calculations, it may not always represent the optimal choice in all imaging contexts.

## 5. Conclusion

The REACT sequence, combined with Adaptive CS-Net reconstruction, enables six-fold accelerated image acquisition with fast reconstruction times, producing images of comparable or superior quality to CS reconstructed mREACT technique and conventional 3D bSSFP. This approach significantly improves image quality for pulmonary veins and upper thoracic and neck vessels, supporting its potential for routine use across diverse patient populations.

## Data Availability

The datasets used and analyzed during the current study are available from the corresponding author upon reasonable request.

## Abbreviations

3D, three-dimensional; AAo, ascending aorta; bSSFP, balanced steady-state free-precession; CEMRA, contrast-enhanced magnetic resonance angiography; CHD, congenital heart disease; CMR, cardiovascular magnetic resonance; CNR, contrast-to-noise ratio; CS, compressed sense; CSA, cross-sectional area; DL, deep learning; ECG, electrocardiogram; LA, left atrium; LPA, left pulmonary artery; LV, left ventricle; MPA, main pulmonary artery; MR, magnetic resonance; MRA, magnetic resonance angiography; MRI, magnetic resonance imaging; PVs, pulmonary veins; RA, right atrium; RPA, right pulmonary artery; REACT, relaxation-enhanced angiography without contrast and triggering; RV, right ventricle; RVOT, right ventricular outflow tract; SVC, superior vena cava.
